# Laparoscopic surgery in 3D improves results and surgeon convenience in sleeve gastrectomy for morbid obesity

**DOI:** 10.1007/s00423-022-02681-8

**Published:** 2022-10-01

**Authors:** Fernando Martínez-Ubieto, Cristian Aragón-Benedí, Ignacio Barranco-Dominguez, Lucía Tardós-Ascaso, Teresa Jiménez-Bernadó, Ana Pascual-Bellosta, José Manuel Ramírez-Rodriguez, Javier Martínez-Ubieto, Research Group in Anesthesia, Resuscitation, Perioperative Medicine of Aragón Health Research Institute (IIS Aragón), Sonia Ortega-Lucea, Jesús Gil-Bona, Luis Alfonso Muñoz-Rodríguez, Guillermo Pérez-Navarro, Natividad Quesada-Gimeno, Berta Perez-Otal, Carmen Heredia-Coca, Jorge Luis Ojeda-Cabrera

**Affiliations:** 1 Department of Bariatric Surgery, Viamed Montecanal Hospital, Cesáreo Alierta 16, 50008 Zaragoza, Spain; 2 Department of Anesthesia, Resuscitation and Pain Therapy, Miguel Servet University Hospital, Zaragoza, Spain; 3 Department of Physiatry and Nursing, Faculty of Health Sciences, University of Zaragoza, Zaragoza, Spain; 4 Department of Surgery, Faculty of Medicine, University of Zaragoza, Zaragoza, Spain

**Keywords:** Laparoscopy, Gastric sleeve, Vertical gastrectomy, 3D image, Bariatric surgery

## Abstract

**Purpose:**

Advanced laparoscopic procedures are still challenging. One critical issue is the lack of stereoscopic vision. The aim of this surgical study is to evaluate whether 3D vision offers any advantages for surgical performance over 2D vision during sleeve gastrectomy for morbid obesity using a laparoscopic system that allows changing between 2D and 3D optics.

**Methods:**

A total of 78 patients were analyzed, with 37 in the 2D group and 41 in the 3D group. Performance time, hospital stay, complications, and early outcomes were collected. To assess the quality of the 2D and 3D techniques, visual analog scales from 0 to 10 were designed, and image quality, depth of field, precision in performing tasks, and general ergonomics were measured.

**Results:**

According to the vision system used, the mean duration of surgery was 85 ± 16.8 min for patients operated on with the 2D system and 69 ± 16.9 min for those operated on with the 3D system. There were no significant differences between the overall percentages of complications according to the type of vision used. However, postoperative complications were more severe in the 2D laparoscopy group. The average length of stay was shorter for patients in the 3D group. Regarding the differences perceived by the surgeon, the depth of field and the precision of tasks were better in the 3D vision group.

**Conclusion:**

The 3D system provided greater depth perception and precision in more complex tasks, enabling safer surgery. This led to a reduction in the operative time and hospital stay. Moreover, the severity of complications was less.

## Introduction

Vertical gastrectomy is currently the most widely used technique in the surgical treatment of obesity and its comorbidities. Its relative technical simplicity and the good results published in medium- and long-term studies have made it the technique of choice in many cases. Nevertheless, possible long-term challenges remain, such as new weight gain and the appearance of de novo gastroesophageal reflux [1–3].

The laparoscopic approach is indicated for this kind of surgery and is usually a two-dimensional approach. However, for some years, the possibility of a three-dimensional approach has come to fruition. Initially, this 3D approach did not gain much popularity since, for most surgeons, undesirable effects such as dizziness, double vision, and instability did not outweigh the potential benefits. However, as these systems have improved, many negative effects have become less prevalent and have thus made the advantages that could be obtained from a 3D vision more apparent, particularly the sense of depth when operating. In short, an improvement in the technique was achieved in the sense that surgeons feel more comfortable and safer during surgery, affecting the operating time and the safety of the surgery [4–6].

Conflicting previous findings on differences between 2 and 3D laparoscopy and the reported lack of studies comparing the use of both methods in bariatric surgery encouraged us to compare various parameters during sleeve gastrectomy, such as image quality, depth of field, precision in performing tasks, and general ergonomics, using a visual analogue scale (VAS). A VAS is a response scale that is widely used in questionnaires. For subjective image quality assessments, VAS provides characteristics that cannot be directly measured. The observer indicates the result making a mark over a 10-cm rule. A VAS could be a better option to determine image quality in endoscopy and radiologic studies. In bariatric surgery, VAS has been used frequently to assess postoperative pain and even hunger and satiety [7, 8], but until now, VAS has been scarcely described in the comparison of 3D and 2D laparoscopic bariatric surgery. The differences that may occur in the comparison of these two types of images in this bariatric surgical technique in terms of the operative time, hospital stay, complications, and postoperative results were assessed in this study using this method with VAS.

The objective of this study is to assess whether 3D laparoscopic surgery applied to the surgical treatment of morbid obesity using the gastric sleeve technique or tubular gastrectomy significantly improves the results of conventional 2D surgery.

## Material and methods

### Study design and setting

A single-center, prospective, observational study was designed that included patients operated on for morbid obesity at Viamed Montecanal Hospital in Zaragoza. Patient recruitment was performed through a consecutive and sequential sampling of cases in a recruitment period from July 2013 and March 2015.

### Ethics

The study was approved by Ethical and Research Committee of Viamed Montecanal Hospital (Zaragoza, Spain), registered No. 2013/21, and written informed consent was obtained from all patients. This study followed the principles of the Declaration of Helsinki by the World Medical Association.

### Patient population and surgery

The data of patients operated on for morbid obesity between July 2013 and March 2015 were collected. The standard 5-trocar tubular gastrectomy or gastric sleeve technique was used, and the surgery was performed by the same surgical team with extensive experience in advanced laparoscopic surgery. Olympus equipment with Endoeye optics (Endoeye Flex 3D/HD, Olympus Winter & IBE GMBH, Hamburg, Germany) was used, which allows the operator to easily change from 2D/HD to 3D/HD vision and vice versa. When in the 3D viewing mode, polarized glasses were used.

The technique in all cases consisted of gastric section from 4 cm of the pylorus to the angle of His on a 42F bougie with Echelon Endoflex of green, golden, or blue loads depending on the thickness of the stomach and at the discretion of the surgeon. The section was oversewn with Surgipro 2/0 in three seromuscular continuous sutures, progressive, starting at the highest part of the sleeve and ending in the antrum. A leak test was performed with methylene blue, and a Jackson-Pratt aspiration drain was left.

### Measurements and data handling

Preoperative comorbidities such as smoking, antiplatelet and anticoagulant intake, history of previous abdominal surgery, heart disease, hypertension (HT), obstructive sleep apnea syndrome (OSA) and the use of nocturnal CPAP devices, diabetes (DM), arthropathy, and dyslipidemia were recorded and studied.

To evaluate the quality of the 2D and 3D techniques, visual analog scales (VASs) from 0 to 10 were designed, and image quality, depth of field, precision in performing tasks, and general ergonomics were measured. We considered for these items 10 as the best definition and 0 as the worst, with 5 being the parameter intermediate.

To measure excess weight loss (EWL) were used Devine’s formula in men ((PI = 50 + 0.91 × (height in cm − 152.4)) and Robinson’s formula in women ((PI = 45.5 + 0.91 × (height in cm − 152.4)).

Postoperative data collection was performed prospectively from the day of surgery until the last review in outpatient consultations up to 2 years after the last surgery. There were no patients lost to follow-up, and only 2 patients changed their place of residence, whose evaluation was completed by telephone.

### Statistical analysis

To perform data analysis, a descriptive analysis was completed using the mean, standard deviation, and quartiles to summarize quantitative data. For qualitative variables, absolute frequency, percentage or relative frequency, and standard error were used together with Chi-squared tests of independence. When inference of quantitative variables had to be tested, the Kruskal–Wallis test and ANOVA tests were used, and a Spearman’s rho correlation test was used to detect the bivariate relationship between variables.

Differences for which the *p* value was < 0.05 were considered significant. The analysis has been developed with R version 3.4.4 (R Foundation for Statistical Computing, Vienna, Austria), and the data were entered into an Excel database (Microsoft Corp., Redmond, WA, USA). The statistical analysis and the review of the data were developed by Jorge Luis Ojeda Cabrera PhD (Department of Statistical Methods of University of Zaragoza).

## Results

### Patient demographic and comorbidities data

During the study period, 78 vertical gastrectomies were performed, 37 using conventional 2D/HD laparoscopy, and 41 using the same technique with the 3D/HD vision system. The groups were homogeneous, and there were no differences between the groups in patient demographic data or comorbidities (Table 1).

**Table 1:** Homogeneity and comparison of demographic data and comorbidities between groups

	2D laparoscopy group *n* = 37		3D laparoscopy group *n* = 41		*p* value
Quantitative variables	Mean	SD	Mean	SD	ANOVA
Age (years)	42.6	11.6	42.1	13.7	0.86
Weight (kg)	118.9	19.1	122.2	21.7	0.47
BMI (kg/m^2^)	43	13.5	42	13.5	0.5
Qualitative variables (*n*)	Percent % (*n*)		Percent % (*n*)		χ2
Male (32)	40.7% (13)		59.3% (19)		0.31
Female (46)	52.2% (24)		47.8% (22)		
Hypertension (25)	35.1% (13)		29.3% (12)		0.57
Diabetes (26)	35.1% (13)		31.7% (13)		0.61
Sleep apnea (25)	37.8% (14)		22% (9)		0.12
Smokers (35)	43% (16)		46% (19)		0.78

Basic descriptives and tests for the demographic and comorbidities data for each group. All variables have no significant relationship with the groups. In other words, for each of these variables, there is no significant difference between the proportion of patients that are assigned to each group. Quantitative variables: mean and standard deviation (SD) for each group, along with comparing mean test (ANOVA). Qualitative variables: quantity or absolute frequency (n), proportion or relative frequency (%, percent) for each group, along with independence tests (Chi2). *Significance defined as *p* value < 0.05. *BMI*, body mass index

The distribution by sex was 46 women (59%) and 32 men (41%) and was similar in both groups. The mean age was 42.3 ± 12.6 years (mean ± SD), with a range between 15 and 70 years, and the age distributions within the groups were very similar.

The mean initial BMI of the series was 42.9 ± 6.33 kg/m^2^, and more than 75% of the patients had a BMI > 39 kg/m^2^. No significant differences were found in the mean BMI of the groups according to the type of vision used (*p* = 0.50) (Table 1).

### Surgical conditions with 2D and 3D technique

In the intraoperative period, the duration of the intervention was 76 ± 18.6 min, with a range between 45 and 120 min. According to the vision system used, the mean duration of the patients operated on with the 2D system was 85 ± 16.8 min, and that of those operated on with the 3D system was 69 ± 16.9 min, a difference that was statistically significant (*p* = 0.0001) (Table 2).

**Table 2:** Perioperative outcomes between groups

	2D laparoscopy group *n* = 37		3D laparoscopy group *n* = 41		Statistical tests	
Quantitative variables	Mean	SD	Mean	SD	*t* test ANOVA	*p* value
Operating time (minutes)	85	16.8	69	16.9	4.26	< 0.0001*
Hospital stay (days)	2.59	0.64	2.15	0.65	2.95	0.0041*
Quantitative variables	Mean	SD	Mean	SD	Spearman’s Rho	*p* value
Depth of field (VAS)	6.89	0.31	8.97	0.15	0.972	< 0.0001*
Precision tasks (VAS)	6.94	0.22	8.97	0.15	0.982	< 0.0001*

Quantitative variables: mean and standard deviation (SD) for each group, along with comparing mean tests (ANOVA or Spearman). *Significance defined as *p* value < 0.05. *VAS*, visual analogue scale

Regarding the differences perceived by the surgeon (Table 2), the depth of field in the 2D vision group obtained an average of 6.89 points on the visual analog scale, while in the 3D vision group, it was 8.97 points. In the precision of tasks, the average points obtained were 6.94 in the 2D group and 8.97 in the 3D group. In both cases, these differences were statistically significant (Spearman Rho 0.97 with *p* < 0.05 and Spearman Rho 0.98 with *p* < 0.05 respectively).

### Postoperative complications with 3D and 2D technique

During the postoperative period, a total of 10 patients (12.8%) suffered complications, 13% of patients in the 2D group, and 12% in the 3D group. In total, there were 14 complications in 10 patients (Table 3). The most frequent complication was fistula in the staple line (*n* = 4, 5.13% of the series), 3 in the 2D group, which caused 1 diffuse peritonitis that required reintervention, and 1 in the 3D group, resolved with conservative treatment. On the other hand, there was 3 sleeve stenosis (*n* = 3, 3.84% of the series), 1 in the 2D group and 2 in the 3D group, resolved with endoscopic dilatation. Finally, there was 3 atelectasis in the 2D group and 1 in the 3D group (4 cases in total, 5.13% of the series), with minimal clinical impairment in all of them (Table 3).

**Table 3:** Complications according to Clavien-Dindo Classification between groups

		2D laparoscopy group *n* = 37	3D laparoscopy group *n* = 41
		*n* (percent %)	*n* (percent %)
Clavien-Dindo Grade I	Atelectasis	3 (8.1%)	1 (2.43%)
Clavien-Dindo Grade II	Malnutrition	0 (0%)	1 (2.43%)
	Guillain–Barre Syndrome	0 (0%)	1 (2.43%)
Clavien-Dindo Grade IIIa	Sleeve Stenosis	1 (2.7%)	2 (4.87%)
Clavien-Dindo Grade IIIb	Fistula	3 (8.1%)	1 (2.43%)
Clavien-Dindo Grade IVa	Peritonitis	1 (2.7%)	0 (0%)
Clavien-Dindo Grade IVb	-	0 (0%)	0 (0%)
Clavien-Dindo Grade V	-	0 (0%)	0 (0%)

There were no significant differences between the overall percentage of complications according to the type of vision used. However, according to the Clavien-Dindo classification [9], the most serious complication (fistula with peritonitis) was more frequent in the 2D laparoscopy group (Spearman Rho − 0.92, *p* = 0.0001).

The average length of stay was used to compare hospital stays. The mean time was 2.59 days in the 2D group and 2.15 days (95% CI) in the 3D group, with the difference being statistically significant and with almost half a day less in the 3D group (− 0.44 days, *p* = 0.0041) (Table 2). No statistically significant differences were found in readmission rates.

### Excess weight loss with 3D and 2D technique

When studying the behavior related to comorbidities after bariatric surgery, we observed improvements in most of them. Approximately 50% of the operated patients stopped taking antihypertensive medication. In the case of arthropathy and OSA and the use of CPAP, both experienced significant postoperative improvements.

Excess weight loss (EWL) at 12 months was very similar in both groups, with 68 ± 18.4% in the 2D vision group and 67 ± 12.8% in the 3D group, with a nonsignificant difference (*p* = 0.66). The same occurred at 24 months, with an EWL of 72.3 ± 18.5% in the 2D group and 71.7 ± 18.2% in the 3D group, with no statistically significant differences (*p* = 0.93). Both systems led to similar weight losses in patients after surgery.

## Discussion

This prospective, observational, cohort study was intended to evaluate the advantages and disadvantages of 3D vision over 2D vision during sleeve gastrectomy. According to the available literature, this article is one of the few studies comparing 2D and 3D laparoscopy in bariatric surgery. Such studies are mainly those carried out by Currò et al. [10] and Martínez-Ubieto et al. [5], which arrived at similar conclusions to those of the present study.

In our study, all patients were operated on by the same surgeon (FMU) and by the same surgical technique, which reduces possible biases derived from the analysis of data obtained with different surgical teams or techniques.

A particularity of the current study was the use of VASs to measure image quality, depth of field, precision in performing tasks, and general ergonomics, since there are no clearly established measurement tools for these parameters in the literature, although there are studies such as the one by Currò et al. that used similar questionnaires [10] and subjective surveys.

### Surgical time between 3D and 2D technique

Other studies, such as those by Wilhelm et al. [11] and Smith et al. [12], both published in 2014, used the validated National Aeronautics and Space Administration Task Load Index (NASA-TLI) workload scale, although the evaluation of the rest of the parameters was carried out with subjective questionnaires. The systematic review carried out in 2017 by the group of Fergo et al. [13] also shows that most of the analyzed studies use subjective parameters.

Regarding the average duration of the surgical intervention, many studies coincide in a reduction of the procedure time using 3D technology when compared to 2D laparoscopy in various types of surgery [14–17]. In the study by Costa et al. in 2021 in colonic surgery [16], the most frequent surgery performed in the world showed a significantly lower anastomotic time (16.9 ± 2.3 min vs. 19.6 ± 2.9 min) in the 3D group compared to the 2D group. More specifically in bariatric surgery, in the study by Padín et al. in 2017 [17], surgical times were also analyzed being 100.22 ± 41.22 min in the 3D group and 124.7 ± 51.97 min in the 2D group, with a statistically significant difference.

In our study, the times were shorter, with 69 ± 16.9 min in the 3D group and 85 ± 16.8 min in the 2D group. Most studies find significant differences between the 2D and 3D techniques, especially in regard to carrying out more complex tasks such as laparoscopic suturing [11, 12, 14].

### Postoperative complications and comorbidities correction

Our incidence of postoperative complications was 12.8%, in line with the Colquitt et al. systematic review [4], where for any type of bariatric surgery, it ranged from 0 to 37%. In addition, mortality in our patient sample was 0%.

There is consensus that the results obtained by bariatric surgery in the correction of comorbidities are much better than those obtained by other medical means, as described by Colquitt et al. in a systematic review of the Cochrane Database of Systematic Reviews in 2014 [4]. Here, a clear improvement in the patient’s comorbidities was observed; practically all of them disappeared (arthropathy, OSA, use of CPAP), while the rest improved significantly (HT, DM), which coincides with that reported in other studies [18–20]. In the specific case of hypertension, in our series, approximately 50% of the operated patients stopped taking antihypertensive medication, coinciding with what was published by Sarkhosh et al. in 2012 in a systematic review, where they found an improvement in hypertension in 75% of patients, achieving complete resolution and the cessation of antihypertensive medication in 58% of cases [21].

### Surgical conditions between 3D and 2D technique

When comparing the 3D and 2D laparoscopy techniques, multiple studies have compared the depth of field perceived by the surgeon as well as the precision in performing more complex tasks such as suturing and knot tying, and the vast majority of the literature favors 3D technology, both in an experimental setting [22, 23] and in human surgery [5, 12, 14]. Some studies have shown a reduction in the number of errors made using 3D laparoscopes compared to classic 2D laparoscopes [13, 17].

When measuring the general ergonomics of laparoscopic surgery with 2D and 3D systems, as previously stated in this discussion, measurement tools are disparate, and subjective evaluations have been used on many occasions. Despite this, most studies find that results in surgeon comfort and adverse effects such as dizziness and headache are the same or improve with 3D systems. When asked about the preference of 2D over 3D, most studies show that experienced and novice surgeons favor 3D surgery [5, 10, 13, 17, 22]. Furthermore, some of these studies have shown a reduced learning curve with 3D laparoscopy systems compared to classic 2D [10, 17, 23].

Currently, there is extensive literature comparing 2D and 3D laparoscopic surgery in various types of interventions. Initially, in the late 1990s, some studies were published that did not show significant differences between the 2D and 3D vision systems, which in addition to not showing significant advantages of 3D systems, found a greater number of adverse effects on the surgeon [24–26]. However, as vision systems have improved, most of the more recent studies comparing these two technologies conclude in favor of the 3D technique for all of the above. In general surgery, studies on laparoscopic cholecystectomy [26, 27], laparoscopic surgery for colon cancer [16, 28, 29], and laparoscopic duodenopancreatectomy [30], as well as previous studies on gastric cancer [31–33] and laparoscopic liver resection [34], reported improvements in surgical time and complications with 3D laparoscopy.

The same is true in other fields, such as gynecology [35], pediatric surgery [36], and urology [37]. However, there are also studies that did not find these differences and established that 3D laparoscopic surgery does not represent advantages over 2D laparoscopic surgery [38, 39].

In 2018, the European Association of Endoscopic Surgery (EAES) published a series of agreed recommendations about 3D surgery. It was confirmed that the surgical time is reduced, as well as the complications, obtaining better results in more complex procedures and making fewer mistakes, although they recommend conducting more studies [40].

In addition, in recent years, more advanced laparoscopy systems are starting to be used with Ultra High Definition such as 2D/4K [41, 42] and even 3D/4K [43] technology, with very favorable results. However, these researchers [44] conclude that the accuracy of conventional 2D/HD laparoscopy devices compared to newer 2D/4K devices are comparable and, as we show, that 3D/HD endovision systems, compared to 2D/HD and 2D/4K, can lead to faster performance and are associated with a lower workload [41, 44].

Therefore, it seems that the advantages of 3D vision systems are clear, especially with regard to the reduction of surgical time, especially in the most complex tasks and in the commission of fewer errors, making surgery safer. In addition, with the implementation of the most modern systems, the disadvantages of dizziness, headaches, and blurred vision that the older systems had have been avoided, so that the advantages far outweigh the disadvantages. However, the price of the systems may be a handicap for their generalization in the operating rooms of all hospitals.

### Limitations of the study

Despite using a convenience sample, one of our main limitations is the small sample of patients used. Besides, our results were based on clinical management under real-life conditions in a single-center. We have not analyzed other factors, such as anesthesia technique and others that are known to contribute to increasing these complications and are probably factors that need to be assessed in subsequent studies and reviews. For all of these reasons, further research is required.

## Conclusion

Thus, based on the results reported by our study, it may be concluded that 3D vision systems in sleeve gastrectomy are accompanied by a reduction in surgical time with fewer postoperative complications and shorter hospital stay. Furthermore, it provides better vision for the surgeon in the depth of field and in the performance of complex tasks.
